# Comparison of the EORTC criteria and PERCIST in solid tumors: a pooled analysis and review

**DOI:** 10.18632/oncotarget.11171

**Published:** 2016-08-10

**Authors:** Jung Han Kim

**Affiliations:** ^1^ Division of Hemato-Oncology, Department of Internal Medicine, Kangnam Sacred-Heart Hospital, Hallym University Medical Center, Hallym University College of Medicine, Seoul 07441, Republic of Korea

**Keywords:** PET, EORTC criteria, PERCIST, tumor response

## Abstract

Two sets of response criteria using PET are currently available to monitor metabolic changes in solid tumors: the criteria developed by the European Organization for Research and Treatment of Cancer (EORTC criteria) and the PET Response Criteria in Solid Tumors (PERCIST). We conducted this pooled study to investigate the strength of agreement between the EORTC criteria and PERCIST in the assessment of tumor response. We surveyed MEDLINE, EMBASE and PUBMED for articles with terms of the EORTC criteria and PERCIST between 2009 and January 2016. We searched for all the references of relevant articles and reviews using the ‘related articles’ feature in the PUBMED. There were six articles with the data on the comparison of the EORTC criteria and PERCIST. A total of 348 patients were collected; 190 (54.6%) with breast cancer, 81 with colorectal cancer, 45 with lung cancer, 14 with basal cell carcinoma in the skin, 12 with stomach cancer, and 6 with head and neck cancer. The agreement of tumor response between the EORTC criteria and PERCIST was excellent (k = 0.946). Of 348 patients, only 12 (3.4%) showed disagreement between the two criteria in the assessment of tumor response. The shift of tumor response between the EORTC criteria and PERCIST occurred mostly in patients with PMR and SMD. The estimated overall response rates were not significantly different between the two criteria (72.7% by EORTC vs. 73.6% by PERCIST). In conclusion, this pooled analysis demonstrates that the EORTC criteria and PERCIST showed almost perfect agreement in the assessment of tumor response.

## INTRODUCTION

The WHO guidelines [1] and Response Evaluation Criteria in Solid Tumors (RECIST) [2] are the most commonly used criteria to assess response to anti-cancer treatment in solid tumors. However, these criteria depending on anatomic changes based on computed tomography (CT) or magnetic resonance imaging (MRI) have limitations in tumors with obscure margins, cystic lesion, or scar tissue. It may be difficult to distinguish necrotic tissue or fibrotic scar from residual tumor by anatomical images [3]. In addition, because these criteria had been developed only for patients receiving cytotoxic treatment, neither of the two criteria has been validated in patients treated with targeted agents that tend to induce necrotic or cystic change, not tumor shrinkage, in solid tumors [4].

Positron emission tomography (PET) with [18F]-fluorodeoxyglucose ([18F]-FDG) has established as a new method for diagnosis and staging of solid tumors [5]. Nowadays, [18F]-FDG PET is also frequently used to monitor tumor responses to anti-cancer therapies. Metabolic changes often occur early in the course of therapy, preceding reduction in the size of tumors. Thus, PET may allow the assessment of tumor response even in the absence of anatomic changes [6–8]. Especially with increasing use of biologically active targeted agent in clinical practice, FDG PET may provide clinicians with more information regarding treatment decision. FDG PET responses have been more significantly correlated with survival than those assessed by CT or MRI [9].

Two sets of response criteria using PET are currently available to monitor metabolic changes to anti-cancer treatment. The European Organization for Research and Treatment of Cancer (EORTC) criteria, the first metabolic criteria for solid tumors, were published in 2000 [10], and the PET Response Criteria in Solid Tumors (PERCIST) was proposed in 2009 [11]. Although the two metabolic criteria have quite different approaches, tumor responses between the two criteria showed almost perfect agreement in several studies with different types of cancers [12–17]. However, each study had a major limitation of a small number of patients. Thus, we conducted this pooled study to investigate the strength of agreement between the EORTC criteria and PERCIST in patients who had received anti-cancer treatment for malignant solid tumors.

## RESULTS

### Eligible studies

There were 6 articles [12–17] in the English literature including the details on the tumor responses according to the EORTC criteria and PERCIST in patients with solid tumors. Two articles [13, 15] compared two metabolic criteria (EORTC vs. PERCIST), and the remaining four studies [12, 14, 16, 17] also compared the tumor responses between morphologic criteria (WHO guidelines and RECIST) and metabolic criteria (EORTC and PERCIST).

### Patients' characteristics

A total of 348 patients with various solid tumors were collected from the six studies; 190 (54.6%) with breast cancer [14, 16, 17], 81 (23.3%) with colorectal cancer [15, 17], 45 (12.9%) with lung cancer [13, 17], 14 with basal cell carcinoma in the skin [12], 12 with stomach cancer [17], and 6 with head and neck cancer [17] (Table 1). One hundred eighty-four patients with breast cancer received neoadjuvant chemotherapy [14, 16], and 81 with colorectal cancer received palliative chemotherapy with or without cetuximab, an epidermal growth factor receptor monoclonal antibody [15, 17]. Fourteen patients with basal cell carcinoma were treated with vismodegib, the first Hedgehog signaling pathway targeting agent [12]. Of 29 patients with small cell lung cancer, 16 had limited disease and 13 extensive disease; they were treated with chemotherapy with or without thoracic radiotherapy [13].

**Table 1:** Summary of six studies comparing the EORTC criteria and PERCIST in malignant solid tumors

Reference	Tumor type	No. of pts	Treatment	kappa value	Discordant rate	Details of discordance	Cause of discordance
						EORTC → PERCIST	
Thacker *et al*. [12]	Basal cell carcinoma	14	Targeted agent (Vismodegib)	1.0	0% (0/14)		No discordance
Ziai *et al*. [13]	Small cell lung carcinoma	29	Chemotherapy or Radiotherapy	1.0	0 % (0/29)		No discordance
Tateishi *et al*. [14]	Breast cancer	142	Neoadjuvant chemotherapy	0.971	1.4% (2/142)	1 SMD → 1 PMR 1 SMD → 1 PMR	10% decrease of SUVmax -> 32% decrease of SULpeak, 13% decrease of SUVmax -> 30% decrease of SULpeak
Skougaard *et al*. [15]	Colorectal cancer	61	Palliative therapy (Irinotecan and cetuximab	0.760	13.1% (8/61)	2 PMR → 2 SMD 4 PMR → 4 SMD 2 SMD → 2 PMR	> 25% decrease of SUVmax -> < 30% decrease of SULpeak Reduction in single-lesion SULpeak was greater than reduction in SUVmax sum, BSA, for multiple lesions. Reduction in single-lesion SULpeak was less than reduction in SUVmax sum, BSA, for multiple lesions.
Tőkés *et al*. [16]	Breast cancer	42	Neoadjuvant chemotherapy	0.951	2.4% (1/42)	1 PMR → 1 SMD	Not available
Aras *et al*. [17]	Colorectal cancer Lung cancer Stomach cancer Head & neck cancer Breast cancer	20 16 12 6 6	Chemotherapy	0.976	1.6% (1/60)	1 PMD → 1 SMD	> 25% increase of SUVmax -> < 30% increase of SULpeak
**Summary**	**Breast cancer Colorectal cancer** **Lung cancer** **Basal cell carcinoma** **Stomach cancer** **Head & neck cancer**	**190** **81** **45** **14** **12** **6**		**0.946**	**3.4%** **(12/348)**	**7 PMR → 7 SMD** **4 SMD → 4 PMR** **1 PMD → 1 SMD**	

Abbreviations: BSA, body surface area; CMR, complete metabolic response; EORTC, European Organization Research and Treatment of Cancer; PERCIST, PET Response Criteria in Solid Tumors; PMD, progressive metabolic disease; PMR, partial metabolic response; SMD, stable metabolic disease; SUV, standardized uptake value; SUL, SUV lean body mass.

### Tumor responses

The rate of discordance in tumor responses between the EORTC criteria and PERCIST was highest (13.1%) in the study of 61 patients with colorectal cancer [15]. Two studies (one with basal cell carcinoma and the other with small cell lung cancer) showed perfect agreement between the two criteria [12, 13]. The comparison of tumor responses according to the EORTC criteria and PERCIST in a total of 348 patients was presented in Table 2. The agreement of tumor response between the two criteria was almost perfect (un-weighted k = 0.946, 95% confidence interval, 0.916–0.976). Of 348 patients, only 12 (3.4%) showed discordance in the assessment of tumor response between the EORTC criteria and PERCIST. The details of the patients showing disagreement were described in Table 1. When adopting the PERCIST, instead of the EORTC criteria, the shift of tumor response occurred mostly in patients with partial metabolic response (PMR) and stable metabolic disease (SMD): 7 patients with PMR according to the EORTC were downgraded to SMD by the PERCIST and 4 with SMD according to the EORTC were upgraded to PMR by the PERCIST. There was only one patient who showed response shift between SMD and progressive metabolic disease (PMD) according the two criteria: from PMD by the EORTC to SMD by the PERCIST. The estimated overall response rates (ORRs), which were estimated in total regardless of the primary tumor sites, were not significantly different between the two criteria (72.7% by the EORTC vs. 73.6% by the PERCIST).

**Table 2:** Comparison of tumor responses according to the EORTC criteria and PERCIST

Tumor response by the EORTC		Tumor response by the PERCIST			Total
	CMR	PMR	SMD	PMD	
CR	70	0	0	0	70
PR	0	179	7	0	186
SD	0	4	38	0	42
PD	0	0	1	49	50
Total	70	183	46	49	348

Abbreviations: CMR, complete metabolic response; EORTC, European Organization Research and Treatment of Cancer; PERCIST, PET Response Criteria in Solid Tumors; PMD, progressive metabolic disease; PMR, partial metabolic response; SMD, stable metabolic disease.

The level of concordance of tumor responses between the EORTC criteria and RECIST is 0.946 (un-weighted k, 95% confidence interval, 0.916–0.976).

The overall response rates were not significantly different between the two criteria (72.7% by the EORTC vs. 73.6% by the PERCIST).

## DISCUSSION

The two metabolic response criteria for PET-based response evaluation, the EORTC criteria and PERCIST, have quite different approaches [10, 11]. The EORTC criteria, the first PET scoring system out in 1999, are based on baseline-chosen, lesion-specific regions of interest (ROIs) that are followed on each subsequent scan [10]. Then standardized uptake value (SUV) is corrected on the basis of body surface area (BSA). In the PERCIST presented 2009, the peak SUV lean body mass (SUL) of hottest single tumor lesion with maximal 12 mm diameter volume ROI (SULpeak) is required in each PET scan [11]. The PERCIST with detailed and unambiguous definitions is considered more uncomplicated to apply in clinical practice than the EORTC criteria [15]. As of now, however, no single method is fully accepted. Because FDG PET is increasingly adopted for response evaluation in clinical trials, it is important to be familiar with the potential differences in the assessment of tumor response using the existing PET response criteria. However, the comparison of tumor responses according to the two criteria has hardly performed in studies with a larger number of patients. In this pooled study, we compared the assessment of tumor responses between the EORTC criteria and PERCIST.

We found that the agreement of tumor responses between the two criteria was almost perfect (k = 0.946). Of 348 patients from the six studies, only 12 (3.4%) showed discrepancy in the assessment of tumor responses between the EORTC criteria and PERCIST. The disagreements between the two criteria were mostly resulted from the differences in the approaches (multiple lesions or single lesion) and in the cutoff values of response (25% or 30%). Especially 6 patients with metastatic colorectal cancer showed discrepancy of tumor response between the two criteria due to the differences in the number of target lesions (multiple lesions in the EORTC criteria vs. single lesion in the PERCIST) [15]. This finding suggests that patients with more metastatic diseases may have higher possibility to show discordance between the two criteria.

In this pooled study, the PERCIST upgraded tumor response in 5 patients and downgraded in 7. The ORRs, which were estimated regardless of the primary tumor sites, were not significantly different between the two criteria (72.7% by the EORTC vs. 73.6% by the PERCIST). When adopting the PERCIST, instead of the EORTC criteria, the shift of tumor response occurred mostly between PMR and SMD. In clinical practice, while patients with PMR or SMD after anti-cancer treatment remain on the same treatment regimen, patients showing PMD usually need to change therapeutic plan. In the current study, there was only one patient who showed response shift between SMD and PMD according to the two criteria. These findings indicate that the clinical impact of exchanging one metabolic response criteria for another may be minimal.

Of note, this pooled study has several limitations. First, patients included in this study were quite heterogeneous in terms of primary sites, clinical setting (neoadjuvant or palliative), and therapeutic regimens. Second, although each study properly followed the criteria to assess tumor response, all studies did not perform FDG PET with the same scanner following the exact same protocol. Third, we could not investigate the prognostic role of the two metabolic criteria. Although the EORTC criteria and PERCIST correlated well with overall survival in two studies [13,15], survival data were not enough to compare prognostic value of the two criteria.

In conclusion, this pooled study demonstrates that the EORTC criteria and PERCIST showed almost perfect concordance in the assessment of tumor response in patients with solid tumors. However, it is still necessary to investigate potential differences between the two criteria in studies with larger homogeneous patients' cohort to elucidate if the criteria can be used interchangeably in clinical practice.

## MATERILAS AND METHODS

### Searching strategy

We searched for all relevant studies written in English through the following searching strategy. A systematic literature search of MEDLINE, PUBMED, and EMBASE from 2009 when the PERCIST were proposed to January 2016 was carried out to find articles including the following terms in their title, abstract, or key words; ‘tumor response’, ‘EORTC criteria’, or ‘PERCIST.’ We also looked into all the references of identified relevant articles and reviews. We used the ‘related articles’ feature of the PUBMED to identify the related articles.

### Study selection criteria

Articles were considered for inclusion in this pooled study if they compared tumor responses by the EORTC criteria and PERCIST. The searched articles were screened by full text review, and the original articles with the details on the assessment of tumor response according to the two criteria were finally included in the study.

### Response categories according to the EORTC criteria and PERCIST

The EORTC criteria normalize SUV using BSA. In each study, the SUVmax values of all target lesions were summed. For assessing tumor responses, the small total SUVmax was subtracted from the large total SUVmax, and the difference was divided by the sum of the SUVmax value from the first PET scan [10]. In the PERCIST, target lesion should be evaluated by the SUL in a maximum of a 12 mm diameter volume ROI in the tumor [11]. The metabolic tumor responses according to the EORTC criteria and PERCIST were described in Table 3.

**Table 3:** Comparison of the EORTC criteria and PERCIST

	EORTC	PERCIST
Complete metabolic response (CMR)	Complete resolution of FDG uptake in all lesions	Complete resolution of FDG uptake in all lesions
Partial metabolic response (PMR)	Greater than 25% reduction in the sum of SUVmax after more than one cycle of treatment	A minimum of 30% reduction of the peak lean body mass SUV (SULpeak) and an absolute drop of 0.8 SULpeak units
Progressive metabolic disease (PMD)	More than 25% increase in the sum of SUVmax or appearance of new FDG-avid lesions	More than 30% increase in the SULpeak of the FDG uptake and an absolute increase of 0.8 SULpeak, or appearance of FDG-avid new lesions
Stable disease (SD)	Not qualify for CMR, PMR or PMD.	Not qualify for CMR, PMR or PMD

Abbreviations: EORTC, European Organization Research and Treatment of Cancer; PERCIST, PET Response Criteria in Solid Tumors.

### Statistical analyses

The ORR was defined as the rate of CMR and PMR. Chi-square test was used to compare the ORRs between two groups, and *P*-value less than 0.05 was considered significant. The level of concordance in tumor responses between the EORTC criteria and PERCIST was estimated using un-weighted k-statistics. The agreement between the two criteria was interpreted as poor (*k* < 0), slight (k = 0–0.20), fair (*k* = 0.21–0.40), moderate (*k* = 0.41 – 0.60), substantial (*k* = 0.61–0.80), and almost perfect (*k* > 0.80).
